# (−)_545_-*fac*-Δ-Tris(l-prolinato)cobalt(III) trihydrate

**DOI:** 10.1107/S1600536808010246

**Published:** 2008-04-18

**Authors:** Masaru Kato, Miho Hayashi, Takashi Fujihara, Akira Nagasawa

**Affiliations:** a Department of Chemistry, Graduate School of Science and Engineering, Saitama University, Shimo-Okubo 255, Sakura-ku, Saitama 338-8570, Japan; b Molecular Analysis and Life Science Center, Saitama University, Shimo-Okubo 255, Sakura-ku, Saitama 338-8570, Japan

## Abstract

The absolute configuration of the octa­hedral *fac*-CoN_3_O_3_ title complex, [Co(C_5_H_8_NO_2_)_3_]·3H_2_O, has been determined by single-crystal X-ray analysis. A three-dimensional network of hydrogen bonds is observed between the proline carboxyl­ate groups and the three uncoordinated water mol­ecules.

## Related literature

For related literature, see: Denning & Piper (1965 ▶).
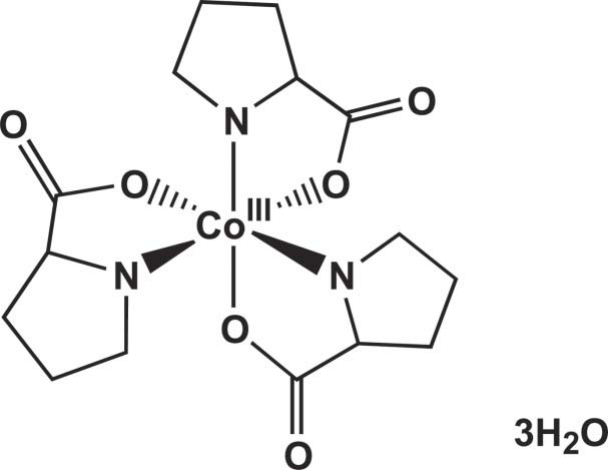


## Experimental

### 

#### Crystal data

[Co(C_5_H_8_NO_2_)_3_]·3H_2_O
*M* *_r_* = 455.35
Orthorhombic, 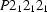

*a* = 10.1673 (9) Å
*b* = 10.8433 (10) Å
*c* = 17.2157 (14) Å
*V* = 1898.0 (3) Å^3^
*Z* = 4
Mo *K*α radiation
μ = 0.96 mm^−1^
*T* = 173 (2) K
0.13 × 0.08 × 0.07 mm

#### Data collection

Bruker SMART APEX CCD area-detector diffractometer
Absorption correction: multi-scan (*SADABS*; Sheldrick, 1996 ▶) *T*_min_ = 0.885, *T*_max_ = 0.936
13762 measured reflections
4522 independent reflections
2969 reflections with *I* > 2σ(*I*)
*R*_int_ = 0.122

#### Refinement

*R*[*F* ^2^ > 2σ(*F* ^2^)] = 0.062
*wR*(*F* ^2^) = 0.107
*S* = 0.92
4522 reflections
274 parameters
H atoms treated by a mixture of independent and constrained refinement
Δρ_max_ = 0.85 e Å^−3^
Δρ_min_ = −0.51 e Å^−3^
Absolute structure: Flack (1983 ▶), 2596 Friedel pairs
Flack parameter: 0.04 (2)

### 

Data collection: *SMART-W2K/NT* (Bruker, 2003 ▶); cell refinement: *SAINT-W2K/NT* (Bruker, 2003 ▶); data reduction: *SAINT-W2K/NT*; program(s) used to solve structure: *SHELXTL-NT* (Sheldrick, 2008 ▶); program(s) used to refine structure: *SHELXTL-NT*; molecular graphics: *ORTEP-3 for Windows* (Farrugia, 1997 ▶); software used to prepare material for publication: *SHELXTL-NT*.

## Supplementary Material

Crystal structure: contains datablocks I, global. DOI: 10.1107/S1600536808010246/tk2254sup1.cif

Structure factors: contains datablocks I. DOI: 10.1107/S1600536808010246/tk2254Isup2.hkl

Additional supplementary materials:  crystallographic information; 3D view; checkCIF report

## Figures and Tables

**Table 1:** Hydrogen-bond geometry (Å, °)

*D*—H⋯*A*	*D*—H	H⋯*A*	*D*⋯*A*	*D*—H⋯*A*
O9—H9*D*⋯O4^i^	0.74 (6)	2.19 (7)	2.907 (6)	164 (8)
O8—H8*D*⋯O2	0.77 (6)	2.11 (6)	2.880 (5)	173 (7)
O7—H7*D*⋯O9^ii^	0.66 (7)	2.20 (6)	2.848 (7)	167 (9)
O9—H9*C*⋯O8	0.77 (6)	2.09 (7)	2.853 (6)	166 (8)
O8—H8*C*⋯O7	0.93 (6)	1.96 (6)	2.882 (7)	172 (5)

Symmetry codes: (i) 
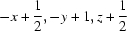
; (ii) 
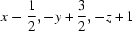
.

**Fig. 1.**
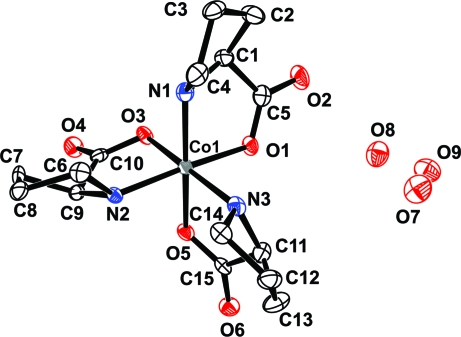
ORTEP drawing for (I), showing atom labelling scheme and displacement ellipsoids at the 50% probability level. All hydrogen atoms are omitted for clarity.

**Fig. 2.**
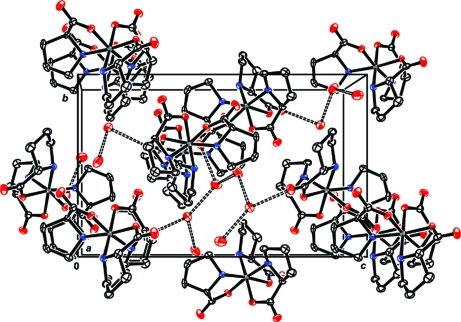
Three-dimensional network structure of hydrogen bonds viewed in projection down the a axis. Dashed lines indicate the hydrogen-bonding interactions. All hydrogen atoms are omitted for clarity.

## supplementary crystallographic information

### Comment

The optical activity, absolute configuration, and rearrangement of tris(*L*-prolinato)cobalt(III) complexes were studied by Denning & Piper (1965), in which the absolute configurations were assigned based on circular dichroism (CD) studies and ^1^H NMR spectra. In this work, single crystals of the (-)_545_-*fac*-[Co(*L*-pro)_3_] isomer (I) suitable for single-crystal X-ray analysis have been prepared. This has allowed the determination of the absolute configuration of (I) as Δ. The UV-Vis and CD spectra of (I) in H_2_O show good agreement with the reported spectra (Denning & Piper, 1965). The molecular structure, Fig. 1, shows three deprotonated proline molecules to chelate the cobalt(III) ion to form octahedral *fac*-Co*N*_3_*O*_3_ geometry. The three lattice water form a three-dimensional network of hydrogen bonds with the uncoordinated carbonyl groups of the proline molecules (Table 1 & Fig. 2).

### Experimental

The title compound was prepared according to the literature method (Denning & Piper, 1965). Single crystals suitable for single-crystal X-ray analysis were obtained by vapor diffusion of ethanol into the aqueous solution of (I) at room temperature. Analysis found: C 39.59, H 6.58, N 9.10; C_15_H_30_CoN_3_O_9_ C_29_H_34_F_6_O_2_S_2_ requires: C 39.57, H 6.64, N 9.23.

### Refinement

The water- and N-bound H atoms were located in difference maps and refined with *U*_iso_(H) = 1.5*U*_eq_(O) or 1.5*U*_eq_(N); see Table 1 for O-H and N-H bond distances. The remaining H atoms were placed in calculated positions, with C—H = 1.00 Å (for CH) and 0.99 Å (for CH_2_) and refined using a riding model, with *U*_iso_(H) = 1.2*U*_eq_(C).

### Crystal data

**Table:**

[Co(C_5_H_8_NO_2_)_3_]·3H_2_O	*F*_000_ = 960
*M**_r_* = 455.35	*D*_x_ = 1.594 Mg m^−^^3^
Orthorhombic, *P*2_1_2_1_2_1_	Mo *K*α radiation λ = 0.71073 Å
Hall symbol: P 2ac 2ab	Cell parameters from 705 reflections
*a* = 10.1673 (9) Å	θ = 2.2–17.1º
*b* = 10.8433 (10) Å	µ = 0.96 mm^−^^1^
*c* = 17.2157 (14) Å	*T* = 173 (2) K
*V* = 1898.0 (3) Å^3^	Block, pink
*Z* = 4	0.13 × 0.08 × 0.07 mm

### Data collection

**Table:**

Bruker SMART APEX CCD area-detector diffractometer	4522 independent reflections
Radiation source: fine-focus sealed tube	2969 reflections with *I* > 2σ(*I*)
Monochromator: graphite	*R*_int_ = 0.122
Detector resolution: 8.366 pixels mm^-1^	θ_max_ = 27.9º
*T* = 173(2) K	θ_min_ = 2.2º
φ and ω scans	*h* = −13→7
Absorption correction: multi-scan(SADABS; Sheldrick, 1996)	*k* = −14→14
*T*_min_ = 0.885, *T*_max_ = 0.936	*l* = −22→22
13762 measured reflections	

### Refinement

**Table:**

Refinement on *F*^2^	Hydrogen site location: inferred from neighbouring sites
Least-squares matrix: full	H atoms treated by a mixture of independent and constrained refinement
*R*[*F*^2^ > 2σ(*F*^2^)] = 0.062	*w* = 1/[σ^2^(*F*_o_^2^) + (0.0283*P*)^2^] where *P* = (*F*_o_^2^ + 2*F*_c_^2^)/3
*wR*(*F*^2^) = 0.107	(Δ/σ)_max_ < 0.001
*S* = 0.92	Δρ_max_ = 0.85 e Å^−^^3^
4522 reflections	Δρ_min_ = −0.51 e Å^−^^3^
274 parameters	Extinction correction: none
Primary atom site location: structure-invariant direct methods	Absolute structure: Flack (1983), 2596 Friedel pairs
Secondary atom site location: difference Fourier map	Flack parameter: 0.04 (2)

### Special details

**Table:**

Geometry. All e.s.d.'s (except the e.s.d. in the dihedral angle between two l.s. planes) are estimated using the full covariance matrix. The cell e.s.d.'s are taken into account individually in the estimation of e.s.d.'s in distances, angles and torsion angles; correlations between e.s.d.'s in cell parameters are only used when they are defined by crystal symmetry. An approximate (isotropic) treatment of cell e.s.d.'s is used for estimating e.s.d.'s involving l.s. planes.
Refinement. Refinement of *F*^2^ against ALL reflections. The weighted *R*-factor *wR* and goodness of fit *S* are based on *F*^2^, conventional *R*-factors *R* are based on *F*, with *F* set to zero for negative *F*^2^. The threshold expression of *F*^2^ > σ(*F*^2^) is used only for calculating *R*-factors(gt) *etc*. and is not relevant to the choice of reflections for refinement. *R*-factors based on *F*^2^ are statistically about twice as large as those based on *F*, and *R*- factors based on ALL data will be even larger.

### Fractional atomic coordinates and isotropic or equivalent isotropic displacement parameters (Å^2^)

**Table:**

	*x*	*y*	*z*	*U*_iso_*/*U*_eq_	
C1	0.3689 (5)	0.9795 (5)	0.1401 (3)	0.0220 (11)	
H1A	0.4364	0.9396	0.1063	0.026*	
C2	0.4210 (5)	1.1046 (5)	0.1661 (3)	0.0309 (15)	
H2A	0.3940	1.1230	0.2201	0.037*	
H2B	0.5182	1.1074	0.1627	0.037*	
C3	0.3589 (5)	1.1948 (4)	0.1093 (3)	0.0248 (11)	
H3A	0.3561	1.2794	0.1309	0.030*	
H3B	0.4062	1.1958	0.0591	0.030*	
C4	0.2218 (5)	1.1408 (5)	0.1008 (3)	0.0251 (12)	
H4A	0.1678	1.1576	0.1475	0.030*	
H4B	0.1766	1.1749	0.0546	0.030*	
C5	0.3343 (5)	0.8910 (5)	0.2056 (3)	0.0216 (12)	
C6	0.0485 (5)	0.7863 (5)	−0.0354 (3)	0.0168 (11)	
H6A	−0.0194	0.7199	−0.0309	0.020*	
C7	0.0427 (5)	0.8403 (5)	−0.1185 (3)	0.0226 (12)	
H7A	0.1316	0.8630	−0.1372	0.027*	
H7B	0.0029	0.7809	−0.1553	0.027*	
C8	−0.0437 (5)	0.9539 (4)	−0.1089 (3)	0.0223 (11)	
H8A	−0.0286	1.0141	−0.1512	0.027*	
H8B	−0.1381	0.9316	−0.1074	0.027*	
C9	0.0024 (5)	1.0038 (5)	−0.0315 (3)	0.0213 (12)	
H9A	0.0893	1.0442	−0.0364	0.026*	
H9B	−0.0614	1.0637	−0.0101	0.026*	
C10	0.1804 (5)	0.7331 (5)	−0.0136 (3)	0.0172 (11)	
C11	−0.0584 (5)	0.8563 (5)	0.2340 (3)	0.0208 (12)	
H11A	−0.0041	0.8448	0.2819	0.025*	
C12	−0.1937 (5)	0.9052 (5)	0.2579 (3)	0.0311 (15)	
H12A	−0.2643	0.8598	0.2305	0.037*	
H12B	−0.2069	0.8960	0.3146	0.037*	
C13	−0.1946 (5)	1.0400 (5)	0.2351 (3)	0.0258 (13)	
H13A	−0.1594	1.0925	0.2773	0.031*	
H13B	−0.2844	1.0681	0.2216	0.031*	
C14	−0.1050 (5)	1.0419 (5)	0.1647 (3)	0.0205 (12)	
H14A	−0.1523	1.0132	0.1178	0.025*	
H14B	−0.0710	1.1261	0.1551	0.025*	
C15	−0.0597 (5)	0.7356 (5)	0.1891 (3)	0.0180 (11)	
Co1	0.12024 (6)	0.87354 (6)	0.11104 (3)	0.01537 (16)	
N1	0.2469 (4)	1.0046 (4)	0.0916 (2)	0.0190 (10)	
H1C	0.2698	0.9957	0.0470	0.023*	
N2	0.0110 (4)	0.8912 (4)	0.01862 (19)	0.0144 (9)	
H2C	−0.070	0.8757	0.0338	0.017*	
N3	0.0039 (4)	0.9561 (4)	0.1851 (2)	0.0194 (11)	
H3C	0.0460	0.9900	0.2108	0.023*	
O1	0.2224 (3)	0.8373 (3)	0.20056 (17)	0.0198 (9)	
O2	0.4133 (3)	0.8715 (4)	0.25896 (18)	0.0321 (9)	
O3	0.2340 (3)	0.7768 (3)	0.04792 (18)	0.0182 (8)	
O4	0.2308 (3)	0.6524 (3)	−0.05483 (18)	0.0275 (9)	
O5	0.0152 (3)	0.7314 (3)	0.12902 (16)	0.0189 (8)	
O6	−0.1273 (4)	0.6473 (3)	0.21062 (17)	0.0246 (8)	
O7	0.1135 (5)	0.9744 (5)	0.4313 (3)	0.0419 (12)	
H7C	0.096 (6)	1.024 (6)	0.392 (4)	0.063*	
H7D	0.051 (7)	0.966 (8)	0.442 (4)	0.063*	
O8	0.2893 (4)	0.7736 (4)	0.3961 (2)	0.0383 (11)	
H8C	0.227 (6)	0.835 (6)	0.404 (3)	0.057*	
H8D	0.328 (6)	0.796 (6)	0.360 (3)	0.057*	
O9	0.3649 (5)	0.5837 (4)	0.5018 (3)	0.0461 (13)	
H9C	0.356 (8)	0.640 (6)	0.475 (4)	0.069*	
H9D	0.328 (7)	0.531 (6)	0.485 (4)	0.069*	

### Atomic displacement parameters (Å^2^)

**Table:**

	*U*^11^	*U*^22^	*U*^33^	*U*^12^	*U*^13^	*U*^23^
C1	0.016 (3)	0.027 (3)	0.023 (2)	−0.002 (3)	0.002 (2)	−0.003 (2)
C2	0.024 (3)	0.034 (4)	0.035 (3)	−0.010 (3)	0.000 (2)	−0.007 (3)
C3	0.029 (3)	0.019 (3)	0.027 (2)	−0.006 (2)	0.009 (3)	−0.002 (3)
C4	0.031 (3)	0.019 (3)	0.025 (3)	0.003 (3)	0.006 (2)	−0.004 (3)
C5	0.028 (3)	0.016 (3)	0.021 (3)	0.005 (2)	0.002 (2)	−0.005 (2)
C6	0.018 (3)	0.018 (3)	0.015 (2)	−0.005 (2)	0.004 (2)	−0.008 (2)
C7	0.024 (3)	0.035 (3)	0.009 (2)	−0.001 (2)	−0.001 (2)	−0.002 (2)
C8	0.019 (3)	0.030 (3)	0.018 (2)	0.000 (2)	−0.001 (3)	0.009 (3)
C9	0.022 (3)	0.018 (3)	0.024 (3)	0.000 (2)	0.000 (2)	0.003 (2)
C10	0.015 (3)	0.017 (3)	0.019 (3)	−0.002 (2)	0.007 (2)	−0.001 (2)
C11	0.028 (3)	0.015 (3)	0.019 (2)	−0.001 (3)	0.003 (2)	0.000 (2)
C12	0.031 (3)	0.025 (4)	0.037 (3)	−0.006 (3)	0.014 (3)	0.001 (3)
C13	0.018 (3)	0.029 (3)	0.030 (3)	0.000 (3)	0.004 (2)	−0.003 (3)
C14	0.022 (3)	0.015 (3)	0.025 (3)	0.003 (3)	−0.003 (3)	0.001 (2)
C15	0.017 (3)	0.018 (3)	0.019 (3)	−0.002 (2)	−0.006 (2)	−0.002 (2)
Co1	0.0161 (3)	0.0153 (3)	0.0147 (3)	−0.0008 (3)	−0.0009 (3)	−0.0009 (3)
N1	0.020 (2)	0.019 (2)	0.018 (2)	0.0016 (19)	−0.0008 (19)	−0.0008 (17)
N2	0.013 (2)	0.015 (2)	0.0159 (19)	0.0001 (19)	−0.0015 (17)	0.0004 (18)
N3	0.026 (3)	0.012 (2)	0.021 (2)	−0.004 (2)	−0.003 (2)	−0.0027 (18)
O1	0.021 (2)	0.023 (2)	0.0158 (17)	0.0023 (17)	−0.0061 (16)	0.0040 (15)
O2	0.033 (2)	0.040 (2)	0.0236 (18)	0.001 (2)	−0.0133 (16)	0.003 (2)
O3	0.0158 (18)	0.019 (2)	0.0199 (17)	0.0037 (16)	−0.0049 (15)	−0.0045 (15)
O4	0.0225 (19)	0.034 (3)	0.0261 (19)	0.0057 (18)	0.0014 (16)	−0.0104 (18)
O5	0.0215 (18)	0.016 (2)	0.0194 (19)	−0.0054 (16)	−0.0042 (15)	−0.0002 (14)
O6	0.0259 (18)	0.021 (2)	0.0270 (17)	−0.005 (2)	−0.0014 (17)	0.0053 (15)
O7	0.041 (3)	0.046 (3)	0.039 (2)	0.009 (3)	−0.003 (3)	0.0153 (19)
O8	0.049 (3)	0.034 (3)	0.032 (2)	−0.001 (2)	0.002 (2)	0.004 (2)
O9	0.048 (3)	0.036 (3)	0.055 (3)	−0.005 (3)	−0.006 (3)	0.004 (2)

### Geometric parameters (Å, °)

**Table:**

C1—N1	1.520 (6)	C11—N3	1.510 (6)
C1—C5	1.521 (7)	C11—C15	1.520 (7)
C1—C2	1.523 (7)	C11—C12	1.530 (7)
C1—H1A	1.0000	C11—H11A	1.0000
C2—C3	1.520 (7)	C12—C13	1.514 (7)
C2—H2A	0.9900	C12—H12A	0.9900
C2—H2B	0.9900	C12—H12B	0.9900
C3—C4	1.518 (6)	C13—C14	1.516 (6)
C3—H3A	0.9900	C13—H13A	0.9900
C3—H3B	0.9900	C13—H13B	0.9900
C4—N1	1.507 (6)	C14—N3	1.488 (6)
C4—H4A	0.9900	C14—H14A	0.9900
C4—H4B	0.9900	C14—H14B	0.9900
C5—O2	1.238 (5)	C15—O6	1.235 (6)
C5—O1	1.282 (6)	C15—O5	1.285 (5)
C6—C10	1.508 (6)	Co1—O1	1.900 (3)
C6—N2	1.518 (6)	Co1—O5	1.900 (3)
C6—C7	1.546 (6)	Co1—O3	1.903 (3)
C6—H6A	1.0000	Co1—N1	1.947 (4)
C7—C8	1.522 (7)	Co1—N2	1.950 (3)
C7—H7A	0.9900	Co1—N3	1.956 (4)
C7—H7B	0.9900	N1—H1C	0.8117
C8—C9	1.513 (6)	N2—H2C	0.8806
C8—H8A	0.9900	N3—H3C	0.7180
C8—H8B	0.9900	O7—H7C	0.88 (6)
C9—N2	1.497 (6)	O7—H7D	0.66 (7)
C9—H9A	0.9900	O8—H8C	0.93 (6)
C9—H9B	0.9900	O8—H8D	0.77 (6)
C10—O4	1.237 (6)	O9—H9C	0.77 (6)
C10—O3	1.282 (5)	O9—H9D	0.74 (6)
			
N1—C1—C5	109.4 (4)	C13—C12—C11	105.7 (4)
N1—C1—C2	106.6 (4)	C13—C12—H12A	110.6
C5—C1—C2	115.2 (4)	C11—C12—H12A	110.6
N1—C1—H1A	108.5	C13—C12—H12B	110.6
C5—C1—H1A	108.5	C11—C12—H12B	110.6
C2—C1—H1A	108.5	H12A—C12—H12B	108.7
C3—C2—C1	103.9 (4)	C12—C13—C14	102.5 (4)
C3—C2—H2A	111.0	C12—C13—H13A	111.3
C1—C2—H2A	111.0	C14—C13—H13A	111.3
C3—C2—H2B	111.0	C12—C13—H13B	111.3
C1—C2—H2B	111.0	C14—C13—H13B	111.3
H2A—C2—H2B	109.0	H13A—C13—H13B	109.2
C4—C3—C2	101.3 (4)	N3—C14—C13	104.5 (4)
C4—C3—H3A	111.5	N3—C14—H14A	110.9
C2—C3—H3A	111.5	C13—C14—H14A	110.9
C4—C3—H3B	111.5	N3—C14—H14B	110.9
C2—C3—H3B	111.5	C13—C14—H14B	110.9
H3A—C3—H3B	109.3	H14A—C14—H14B	108.9
N1—C4—C3	103.5 (4)	O6—C15—O5	123.0 (5)
N1—C4—H4A	111.1	O6—C15—C11	121.3 (4)
C3—C4—H4A	111.1	O5—C15—C11	115.8 (4)
N1—C4—H4B	111.1	O1—Co1—O5	90.41 (14)
C3—C4—H4B	111.1	O1—Co1—O3	90.97 (14)
H4A—C4—H4B	109.0	O5—Co1—O3	89.27 (14)
O2—C5—O1	123.3 (5)	O1—Co1—N1	85.92 (15)
O2—C5—C1	120.5 (5)	O5—Co1—N1	172.63 (16)
O1—C5—C1	116.2 (4)	O3—Co1—N1	84.41 (15)
C10—C6—N2	111.0 (4)	O1—Co1—N2	173.64 (17)
C10—C6—C7	114.1 (4)	O5—Co1—N2	83.85 (15)
N2—C6—C7	105.9 (4)	O3—Co1—N2	86.24 (15)
C10—C6—H6A	108.6	N1—Co1—N2	99.48 (16)
N2—C6—H6A	108.6	O1—Co1—N3	84.07 (15)
C7—C6—H6A	108.6	O5—Co1—N3	85.70 (16)
C8—C7—C6	103.2 (4)	O3—Co1—N3	172.90 (16)
C8—C7—H7A	111.1	N1—Co1—N3	100.25 (17)
C6—C7—H7A	111.1	N2—Co1—N3	98.17 (16)
C8—C7—H7B	111.1	C4—N1—C1	104.8 (4)
C6—C7—H7B	111.1	C4—N1—Co1	125.9 (3)
H7A—C7—H7B	109.1	C1—N1—Co1	108.3 (3)
C9—C8—C7	101.9 (4)	C4—N1—H1C	105.4
C9—C8—H8A	111.4	C1—N1—H1C	105.4
C7—C8—H8A	111.4	Co1—N1—H1C	105.4
C9—C8—H8B	111.4	C9—N2—C6	105.8 (3)
C7—C8—H8B	111.4	C9—N2—Co1	125.7 (3)
H8A—C8—H8B	109.3	C6—N2—Co1	106.5 (3)
N2—C9—C8	103.6 (4)	C9—N2—H2C	105.8
N2—C9—H9A	111.0	C6—N2—H2C	105.8
C8—C9—H9A	111.0	Co1—N2—H2C	105.8
N2—C9—H9B	111.0	C14—N3—C11	105.5 (4)
C8—C9—H9B	111.0	C14—N3—Co1	125.6 (3)
H9A—C9—H9B	109.0	C11—N3—Co1	106.8 (3)
O4—C10—O3	124.0 (5)	C14—N3—H3C	105.8
O4—C10—C6	119.8 (5)	C11—N3—H3C	105.8
O3—C10—C6	116.2 (4)	Co1—N3—H3C	105.8
N3—C11—C15	109.7 (4)	C5—O1—Co1	116.5 (3)
N3—C11—C12	106.2 (4)	C10—O3—Co1	114.7 (3)
C15—C11—C12	115.3 (4)	C15—O5—Co1	115.8 (3)
N3—C11—H11A	108.5	H7C—O7—H7D	95 (7)
C15—C11—H11A	108.5	H8C—O8—H8D	104 (6)
C12—C11—H11A	108.5	H9C—O9—H9D	108 (8)

### Hydrogen-bond geometry (Å, °)

**Table:**

*D*—H···*A*	*D*—H	H···*A*	*D*···*A*	*D*—H···*A*
O9—H9D···O4^i^	0.74 (6)	2.19 (7)	2.907 (6)	164 (8)
O8—H8D···O2	0.77 (6)	2.11 (6)	2.880 (5)	173 (7)
O7—H7D···O9^ii^	0.66 (7)	2.20 (6)	2.848 (7)	167 (9)
O9—H9C···O8	0.77 (6)	2.09 (7)	2.853 (6)	166 (8)
O8—H8C···O7	0.93 (6)	1.96 (6)	2.882 (7)	172 (5)

Symmetry codes: (i) −*x*+1/2, −*y*+1, *z*+1/2; (ii) *x*−1/2, −*y*+3/2, −*z*+1.
